# Post-market surveillance of albendazole tablet brands in Kenya: Implications for deworming programs and clinical outcomes

**DOI:** 10.1371/journal.pntd.0014449

**Published:** 2026-06-16

**Authors:** Ezra Kiprono Yator, Peter Mbugua Njogu

**Affiliations:** University of Nairobi, Faculty of Health Sciences, Department of Pharmaceutical Chemistry, Pharmaceutics & Pharmacognosy, Nairobi, Kenya; Bayer AG, SWITZERLAND

## Abstract

**Background:**

Albendazole is a broad-spectrum anthelminthic extensively used in clinical settings and national deworming programs. It is the cornerstone of the preventive chemotherapy for soil-transmitted helminthiases prevalent in low-resource settings. The high proliferation of albendazole generic products, with poor post-market surveillance capacity in the low- and middle-income countries, poses dire risk of substandard and falsified medicines, which may predispose patients to treatment failures, adverse drug reactions, morbidity, and mortality, with consequent loss of public confidence in healthcare systems.

**Objective:**

This study aimed to determine the quality and pharmaceutical equivalence of albendazole 400 mg tablet brands marketed in Nairobi, Kenya.

**Experimental:**

A cross-sectional analytical study was conducted on seven albendazole 400 mg tablet brands purchased from pharmacy outlets in Nairobi. Tests for identity, friability, hardness, disintegration, assay, uniformity of weight, and dissolution were conducted as specified in the United States, British and International Pharmacopoeias. Dissolution profiles of generic albendazole tablet brands and the innovator brand (Zentel) were compared using model independent fit factors *f*_*1*_ and *f*_*2*_, and the dissolution efficiency (DE). Data was captured and analyzed using Microsoft Excel 2021, and reported as means, relative standard deviations, and percentages.

**Results:**

All the seven albendazole 400 mg tablet brands complied with compendial specifications for identity, friability, hardness, assay, and uniformity of weight. However, two brands (EK5 and EK6) did not comply with the disintegration test and consequently demonstrated extremely poor dissolution, having released <6% of labelled albendazole content at 60 minutes with DE < 5%. Only two brands (EK4 and EK2) exhibited dissolution profiles that approximated the innovator brand (*f*_*2*_ > 50, *f*_*1*_ *<* 10) and could be used interchangeably, while the other two (EK3 and EK7) had intermediate drug release.

**Conclusion:**

While all tested albendazole 400 mg tablet brands complied with basic pharmacopoeial specifications for quality, dissolution testing revealed significant nonequivalence, with only three (42.86%) of the seven brands being pharmaceutically equivalent. Poor dissolution of majority (57.14%) of albendazole tablet brands portends therapeutic insufficiency and development of drug resistance, reinforcing the need for stringent post-market surveillance to safeguard public health.

## Author summary

Albendazole is an important medicine for worm infections. Several generic albendazole products are readily available, and due to their affordability, have improved access to treatment. Nevertheless, generics should meet the same standards of quality, safety, and efficacy as the innovator (originator) product to guarantee equitable access to healthcare. In this study we evaluated seven albendazole 400 mg tablet brands sold in Nairobi City to determine whether they comply with regulatory criteria for quality and are likely to be effective in the body. All seven albendazole tablet brands met the tests for drug content, uniformity of weight, and hardness, implying that none of them was a falsified medicine. However, only three (42.9%) brands met the requirement for dissolution, indicating that more than half of the tested brands may not achieve the desired treatment. Two brands completely failed to disintegrate and released <5% of the medicine. The findings imply that routine tests may not guarantee the quality and effectiveness of albendazole tablet brands, creating a compelling need for stringent regulatory oversight such as post-marketing surveillance. This should necessarily involve disintegration and dissolution testing to ensure that albendazole tablet brands approved for use continue to protect vulnerable communities and deliver desired health benefits.

## Introduction

Soil-transmitted helminthiases (STH) are among the most prevalent neglected tropical diseases (NTDs), afflicting about a quarter of the global human population [1]. Approximately 85% of the NTD disease burden is attributable to STH [2], with estimated 1.38 million disability-adjusted life years (DALYs) lost to STH annually [3]. The impact of STH on the global burden of disease is most severe in the low- and middle-income countries (LMICs) where access to proper sanitation and hygiene is severely constrained [4]. Hundreds of millions of school-aged children in endemic zones are vulnerable to helminthic infections [5,6], contributing to substantial disease burden in low-resource settings with consequent heavy healthcare costs that retard socioeconomic development of the affected individuals and communities. Hence, the deleterious effects of these parasitic infections transcend mere clinical pathology. Their socioeconomic impact extends to cognitive impairment, reduced educational achievement, and perpetuation of generational poverty among endemic populations [7].

**Fig 1:**
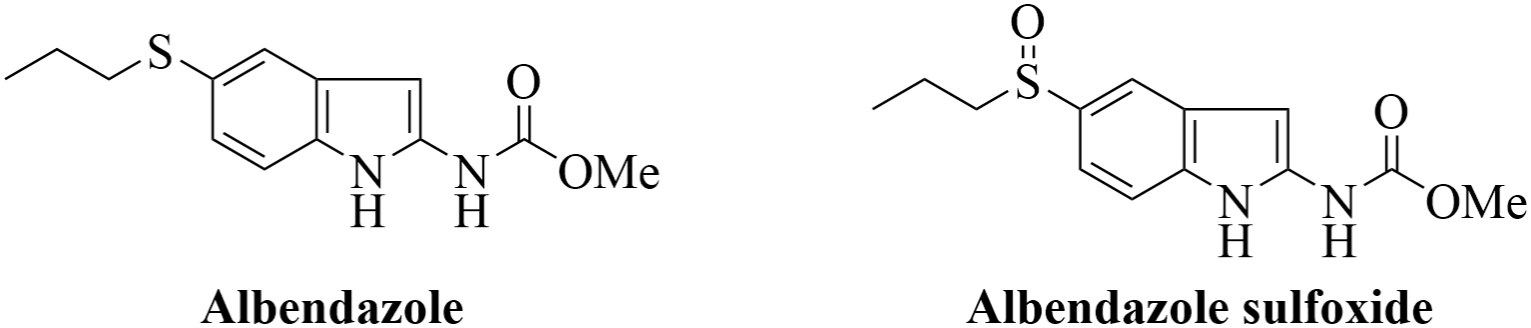
Chemical structures of albendazole and albendazole sulfoxide.

Albendazole (Fig 1), a benzimidazole carbamate derivative, is one of the foremost anthelminthic drugs globally. It has a broad-spectrum coverage against roundworms, hookworms, and tapeworms, making it a linchpin anthelminthic in community- and school-based deworming programs in endemic regions [8,9], and the first-line treatment for hydatid disease [10]. It is particularly preferred because of its convenience of administration as a single bolus dose for most helminthic infections. Albendazole exerts anthelminthic effect through albendazole sulfoxide that is generated in vivo through first-pass metabolism. Albendazole sulfoxide binds to and prevents polymerization of the helminthic β-tubulin [11], inhibiting cytoplasmic microtubule formation and glucose uptake within the parasite, resulting in immobilization and death of adult worms. It also prevents parasite eggs from hatching [12].

The proliferation of generic albendazole products in the LMICs, including Kenya, presents a critical regulatory challenge. Global estimates indicate that approximately one in ten medicines circulating in LMICs are substandard or falsified (SF) products [13], an immense concern for anti-infectives where subtherapeutic exposure accelerates the emergence and spread of antimicrobial and antiparasitic drug resistance [14]. National Medicines Regulatory Authorities (NMRAs) require generic products to demonstrate pharmaceutical equivalence with innovator brands, yet post-market surveillance and pharmacovigilance infrastructure in the LMICs is acutely under-resourced and consequently hampered by severe operational and technological deficits [15].

Several post-market surveillance studies conducted across different African countries accentuate major gaps in quality assurance of critical medicines in the continent. For example, a review of anthelmintics in the East Africa region conducted by Tegegne et al. in 2024 reported that 56% of the analyzed products were noncompliant with compendial specifications [16]. Two other studies, one each in Tanzania and Ethiopia, highlight the same deficiencies. While the Tanzanian study by Kimaro et al. [17] showed that only 25% of albendazole tablets met dissolution requirement, the Ethiopian study by Mekasha et al. [18] found that none of the six analyzed albendazole tablet brands were compliant with this specification. Such quality inadequacies do not augur well for therapeutic efficacy of albendazole tablets and pose the risk of development of drug resistant helminthes [19].

With a calculated Log P value of 3.85 and topological polar surface area of only 50.36 Å^2^, albendazole is a classical high lipophilicity and low aqueous solubility molecule. Based on the Biopharmaceutics Classification System (BCS) [20], albendazole is a BCS Class II drug where dissolution rate governs *in vivo* absorption. Consequently, small variations in formulation composition or manufacturing processes can produce clinically significant non-equivalence. The present study addresses a critical surveillance gap by comprehensively evaluating the quality and pharmaceutical equivalence of albendazole 400 mg tablet brands marketed in Nairobi City County, the central pharmaceutical distribution hub for Kenya and the East African region.

## Experimental

### Study site

This study was conducted in the Drug Analysis and Research Unit (DARU) at the School of Pharmacy, University of Nairobi, Kenya.

#### Study design.

A cross-sectional laboratory-based analytical experiment of seven albendazole 400 mg tablet brands, comprising the originator product and six generic formulations with marketing authorization in Kenya, was conducted. With reference to general and specific monographs for albendazole 400 mg tablets in the International Pharmacopoeia (Ph. Int.) [21], British Pharmacopoeia (BP) [22], and United States Pharmacopeia (USP) [23], the seven tablet brands were subjected to compendial tests for identity, uniformity of weight, tablet hardness, friability, disintegration, assay for the active pharmaceutical ingredient (API), and dissolution. Further, using dissolution profile comparison, the seven brands were evaluated for pharmaceutical equivalence.

#### Study area.

The research was conducted in Nairobi City County, Kenya, which serves as a central hub for drug distribution in Kenya and the East African Community (EAC) region.

#### Sampling technique.

For each of the seven albendazole 400 mg tablet brands, 100 tablets were purchased from various retail and wholesale pharmacies in Nairobi City County by convenience sampling, using the mystery shopper approach.

#### Materials, reagents and equipment.

The seven albendazole 400 mg tablet brands were purchased in their original packaging and coded alphanumerically (EK1–EK7), with EK1 being the innovator brand. Analytical grade sodium hydroxide pellets, concentrated hydrochloric acid, and methanol (Sigma Aldrich) were used. Albendazole chemical reference substance (CRS) was provided by the Drug Analysis & Research Unit. The test for identity and the assay for albendazole were conducted using a Genesys 10S Ultraviolet-Visible (UV-Vis) spectrophotometer (Thermo Scientific, Madison, WI, USA). Friability testing was conducted on a EF2 Electrolab friabilator (Heusenstamn Kr. Offenbach/Main, Germany) while hardness testing was conducted using a Schleuniger-2E Electronic hardness tester (Dr. K.Schleuniger and Co, Switzerland). Disintegration was conducted on an Erweka-Apparatebau-G.m.b.H. disintegration tester (Heusenstamn Kr. Offenbach/Main, Germany), while dissolution testing was performed using an Electrolab dissolution tester (Electrolab India Pvt. Ltd, Mumbai, India) fitted with type 2 apparatus. All weight measurements were taken on a Shimadzu AUW220D electronic semi-micro analytical weighing balance (Shimadzu Corporation, Kyoto, Japan).

#### Preparation of the standard curve.

A 10.0 mg aliquot of albendazole CRS was weighed and dissolved in 0.1 M methanolic HCl in a 100 mL volumetric flask to obtain a 100 µg/mL stock solution. Aliquots of 2.0, 3.0, 4.0, 5.0, and 6.0 mL of this stock solution were transferred into 50 mL volumetric flasks and made to volume with 0.1 M NaOH to yield solutions of 4, 6, 8, 10, and 12 µg/mL albendazole, respectively, and their UV absorbances measured in triplicate at 308 nm against 0.1 M NaOH blank. A calibration curve was prepared by plotting a chart of mean absorbance against albendazole concentration.

#### Analytical procedures.

##### Test for identity:

The identity of albendazole active pharmaceutical ingredient (API) in the analyzed albendazole 400 mg tablet brands was determined by ultra-violet/visible (UV) spectrophotometry. The analyte UV spectra were compared with the UV spectrum of albendazole CRS obtained at the same time as specified in the Ph. Int. (2025) monograph for albendazole chewable tablets.

##### Test for weight uniformity:

The collective weight of 20 random albendazole 400 mg tablets from each brand was determined, and the average weight calculated. The tablets were then individually weighed, and the variation from the mean weight calculated, expressed as the relative standard deviation (RSD), and the tablets evaluated for compliance with the BP (2024) specification for weight uniformity.

##### Test for mechanical strength:

Tablet hardness was conducted according to the USP (2025) guidance on crushing strength. For each brand, six albendazole 400 mg tablets were used. Each tablet was placed between the jaws of an electronic hardness tester oriented similarly to the direction of force application. The mechanical force was gradually increased, the strength at which tablet fracture occurred recorded, and the average mechanical strength calculated.

##### Friability test:

Separately for each brand, 20 albendazole 400 mg tablets were weighed, placed in the friabilator, and tumbled at 25 revolutions per minute (rpm) for four minutes. The tablets were then dusted, re-weighed, and the weight loss calculated. The percentage friability was compared to the BP (2024) acceptance criteria.

##### Disintegration test:

For each brand, six albendazole 400 mg tablets were placed independently in six baskets of the disintegration tester and lowered into a one-litre vessel containing 900 mL of 0.1 N HCl maintained at 37 ± 1°C. The time taken for complete disintegration of each tablet was recorded, and the average time compared to the BP (2024) specifications.

##### Assay for albendazole:

The assay for albendazole was carried out according to the Ph. Int. (2025) specifications for albendazole chewable tablets. Twenty albendazole 400 mg tablets were weighed and pulverized to a fine powder. To a quantity of powder containing 0.40 g of albendazole in a 100 mL volumetric flask, 60 mL of 0.1 M methanolic HCl was added, shaken for 15 minutes to dissolve, the solution made to volume with 0.1 M methanolic HCl, and filtered. To a 2.0 mL aliquot of the filtrate, 0.1 M NaOH was added to dilute to 100.0 mL, the absorbance of the resultant solution measured at 308 nm, and the content of albendazole calculated using the linear regression equation obtained in the calibration curve.

##### Dissolution testing and pharmaceutical equivalence:

Dissolution testing was carried out according to the USP (2025) method, using 900 mL of 0.1 N HCl maintained at 37 ± 0.5 °C, and dissolution apparatus 2 rotating at 75 rpm. For each of the seven albendazole 400 mg tablet brands, six tablets were randomly selected, placed individually in separate vessels, and subjected to dissolution for 60 minutes. At 5, 15, 30, 45, and 60 minutes, 5.0 mL aliquots were sampled and replenished with an equal volume of fresh dissolution medium to maintain sink conditions. The solutions were filtered and assayed by UV spectrophotometry at 308 nm. The percentage of the labelled content of albendazole released at 30 minutes was used to determine compliance with the pharmacopoeial specification for dissolution testing. Pharmaceutical equivalence was evaluated by comparing the dissolution profiles of the six generic formulations to that of the innovator brand over the 60 minutes using the difference factor (*f₁*), similarity factor (*f₂*), and dissolution efficiency (DE).

#### Data management and statistical analysis.

The data generated was recorded in an Excel (Microsoft Office LTSC Standard 2021) spreadsheet for data analysis, and the results archived digitally. The data for uniformity of weight, friability, tablet hardness, disintegration, assay, and dissolution testing were tabulated, while the dissolution profiles were represented graphically. The assay content calculation was based on the linear regression equation for the calibration graph, while the difference factor (*f₁*), similarity factor (*f₂*) and dissolution efficiency (DE) were computed from the dissolution data to infer pharmaceutical equivalence as previously described [24,25].

## Results

### Calibration curve for quantitation of albendazole

**Fig 2:**
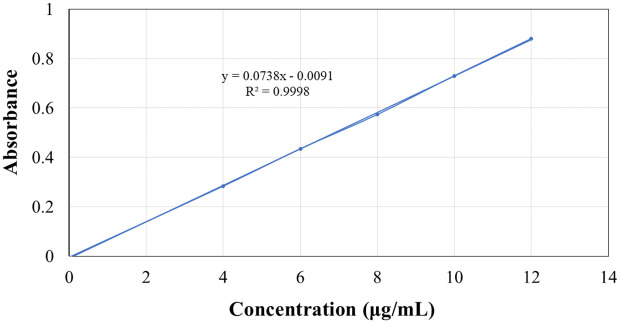
Calibration curve for albendazole obtained by UV/Vis spectrophotometry.

The linear regression equation for the albendazole calibration curve was y = 0.0738x-0.0091 (Fig 2). The high coefficient of determination (R^2^ = 0.9998) showed a strong linear relationship between absorbance and the concentration of albendazole over the 0–12 µg/mL concentration range.

### Compendial quality parameters

The results of weight uniformity, hardness, friability, assay and disintegration tests are summarized in Table 1. All the seven albendazole 400 mg tablet brands (EK1-EK7) complied with the Ph. Int. (2025) test for identity by UV spectrophotometry, confirming the presence of albendazole as the API in all the seven brands and ruled out counterfeit substitution.

**Table 1:** Quality control test results of the analyzed albendazole tablet brands.

Brand code	Average weight in grams (SD) n = 20	Deviation from average weight (%)	Hardness (N) average (SD)	Friability (%)	Assay (%) (RSD)	Disintegration time in min (SD); n = 6
EK1	1.0288 (0.0079)	-1.4234 – 1.4147	161.17 (7.47)	0.08	103.1 (0.2102)	5.01 (0.42)
EK2	0.9970 (0.0146)	-2.2186 – 2.0341	171.67 (4.82)	0.22	105.3 (0.8554)	3.95 (0.18)
EK3	0.7508 (0.0294)	-5.4742 – 9.4433	109.67 (5.37)	0.27	99.5 (0.0573)	1.99 (0.57)
EK4	1.0509 (0.0266)	-3.9246 – 4.2395	107.50 (13.70)	0.23	103.1 (1.6552)	2.78 (0.18)
EK5	0.6865 (0.0157)	-7.8852 – 3.2140	120.17 (37.39)	0.25	95.3 (1.6102)	> 60
EK6	0.9780 (0.0119)	-1.6642 – 3.5098	162.67 (19.69)	0.18	103.1 (1.2735)	> 60
EK7	0.7863 (0.0072)	-2.6531 – 1.9383	200.00 (0.00)	0.13	100.8 (1.3413)	7.07 (0.46)

EK1, Innovator brand; RSD, Relative standard deviation; SD, Standard deviation.

Weight uniformity was acceptable across all brands, with deviations ranging from -7.8852% to +9.4433%. Although some individual tablets in Brands EK3 and EK5 approached the upper limits, they remained within the BP (2024) tolerance of not more than two tablets exceeding ±5% deviation from the average tablet weight for tablets weighing more than 250 mg. Brand EK3 had two tablets deviating from the mean by more than ±5%. No tablet deviated by more than ±10% from the average tablet weight. This suggests overall adequate process control in all the tested albendazole 400 mg tablet brands, in line with Good Manufacturing Practice (GMP) requirements.

The hardness test indicates how resistant a tablet is to mechanical stress during packaging, transport, and handling. It also correlates with friability and disintegration performance. All the tablet dimensions varied by less than 0.01 cm, so hardness was reported directly as breaking force (N) rather than tensile strength. The values ranged from 107.5 N (EK4) to 200 N (EK7). Brand EK5 showed high variability (SD = 37), suggesting inconsistency in manufacturing. Brand EK7’s 200.0 N with SD = 0 represented an upper-limit ceiling set by the hardness tester, not true zero variability. The extremely high hardness for Brand EK7 indicates excessive compression force or low lubricant concentration, leading to dense tablets with low porosity. Such hardness can impede water penetration, slowing disintegration and subsequent dissolution. All the seven albendazole 400 mg tablet brands met the acceptance criteria (USP 2025) for crushing strength, since all values exceeded 40 N. The acceptable tablet hardness values suggest acceptable die fill and compression force during manufacture.

Friability (0.08-0.27%) was less than 1% for all the seven albendazole 400 mg tablet brands, complying with the BP (2024) specification for friability, suggesting sufficient binding capacity in the formulations. This test is a measure of tablet resistance to surface abrasion and predicts potential behaviour under handling and packaging stress.

The assay results for albendazole, based on the linear regression equation from the calibration curve, i.e., y = 0.0738x-0.0091, revealed that all brands had drug content within the 90–110% limits, complying with the Ph. Int. (2025) specifications. Brand EK2 had the highest content (105.3%) while Brand EK5 had the least content (95.3%). The high percentage content in Brand EK2 likely reflects an overage policy to get an edge against long-term degradation. The low RSD values across the brands indicate acceptable content uniformity within batches.

Except for two brands (EK5 and EK6), disintegration times for the other five brands ranged from 1.99 min (EK3) to 7.07 min (EK7) complying with the British Pharmacopoeial limit of 15 minutes for film-coated tablets (Table 1). Brand EK3 disintegrated the fastest (1.99 min), suggesting higher porosity and possibly the inclusion of efficient disintegrants. Conversely, the disintegration of Brand EK7 was much slower (7.07 min), consistent with its high hardness and reduced porosity, resulting in reduced water ingress. Of great concern were the Brands EK5 and EK6 that completely failed to disintegrate within the pharmacopoeial time limit. Since the two brands had relatively moderate hardness, the failure to completely disintegrate could be attributed to formulation failure, for example, due to the omission of disintegrants or over-lubrication. The failure of these brands to comply with disintegration test foreshadowed their abysmal dissolution performance.

### Dissolution testing

Dissolution testing revealed significant differences among the seven albendazole 400 mg tablet brands (Table 2). The innovator Brand EK1 demonstrated rapid dissolution, releasing approximately 94% of the labelled content (Q) within the specified 30 minutes. Brands EK2, EK4 and EK7 exhibited a similar dissolution trend, attaining 88.4%, 97.7%, and 80.6%, respectively. The four brands (EK1, EK2, EK4, and EK7) therefore satisfied the dissolution requirement of not less than 80% of drug release within 30 minutes, in compliance with USP (2025) Dissolution Test 2 specification for albendazole tablets. Brand EK3 achieved moderate dissolution, with approximately 79% of labelled content released at 30 minutes, barely missing the USP acceptance criterion. Nevertheless, at 45 min, it was among the five albendazole 400 mg tablet brands (EK1, EK2, EK3, EK4 and EK7) that had released more than 80% Q, indicating potential therapeutic interchangeability in clinical settings [26].

**Table 2:** Dissolution data, fit factors *f*_1_ and *f*_2_, and dissolution efficiency of albendazole tablet brands.

	Average % albendazole released (n = 6)					Fit factors		Dissolution efficiency	
Brand code	5 min	15 min	30 min	45 min	60 min	*f*_*1*_	*f*_*2*_	AUC	DE
EK1	48.543	84.790	94.219	98.058	99.554	–	–	5054.75	84.25
EK2	48.910	79.596	88.432	92.638	90.803	6.00	63.53	4758.84	79.31
EK3	33.525	63.674	78.551	85.806	89.956	17.32	42.79	4187.40	69.79
EK4	48.120	91.960	97.663	99.977	99.469	3.07	72.85	5221.02	87.02
EK5	1.993	2.981	3.630	4.421	5.268	95.70	5.94	212.48	3.54
EK6	2.275	2.840	3.179	3.179	4.251	96.30	5.78	179.81	3.00
EK7	29.376	64.888	80.612	84.564	88.262	18.22	41.91	4171.03	69.52

AUC, Area under the curve; DE, dissolution efficiency

In contrast, Brands EK5 and EK6 performed dismally, releasing only 3.6% and 3.2% of albendazole at 30 min, that rose marginally to 5.3% and 4.3%, respectively, at 60 min. The two brands were non-compliant with compendial specifications for dissolution testing and are evidently pharmaceutically non-equivalent to the innovator brand and should therefore not be used interchangeably in clinical settings. Their poor performance can be attributed to formulation failure, but not limited to excessive compaction pressures, absence of disintegrants and over-lubrication. They are unlikely to achieve effective therapeutic concentration *in vivo*, leading to therapeutic failure and propagating drug resistance.

### Pharmaceutical equivalence

**Fig 3:**
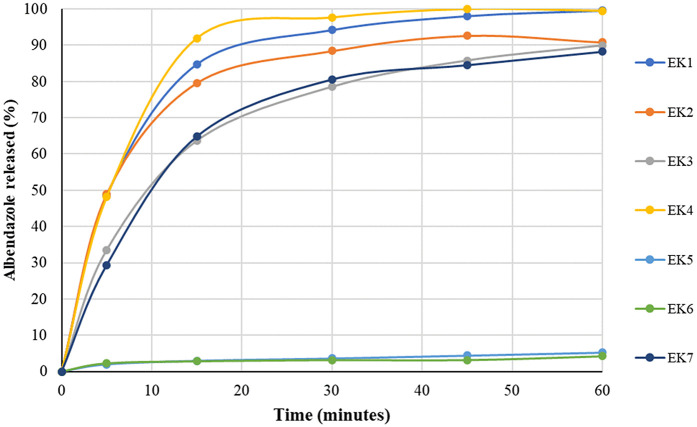
Dissolution profiles of the analyzed albendazole 400 mg tablet brands.

The comparative dissolution profiles of the seven albendazole 400 mg tablet brands analyzed in this study are graphically represented in Fig 3. Brand EK4 had an *f*_*2*_ of 72.85 and an *f*_*1*_ of 3.07 relative to the innovator Brand EK1, hence satisfying the acceptance criteria for pharmaceutical equivalence. Additionally, Brand EK2, with an *f*_*2*_ of 63.53 and *f*_*1*_ of 6.00, met equivalence thresholds. This suggests adequate formulation strategies that mirror the innovator brand. On the other hand, Brands EK3 and EK7 had *f*_*2*_ values of 42.79 and 41.91 respectively, which fell short of the specified EMA thresholds, while their *f*_*1*_ values (17.32 and 18.22, respectively) exceeded the 0–15 limit. These two profiles, though compliant with the pharmacopoeial requirement for drug release at specified time points, demonstrated kinetic release dissimilarity, and are therefore not truly pharmaceutical equivalent to the innovator brand. On the other hand, Brands EK5 and EK6 had *f*_*1*_ values less than 6 and *f*_*2*_ values exceeding 95, reflecting strong dissimilarity with the reference innovator brand. The two brands are therefore starkly pharmaceutically nonequivalent to the innovator brand and are unlikely to achieve intended therapeutic efficacy.

Dissolution efficiency (DE) is a function of the rate and extent of drug release within a defined period, which in this case was 60 minutes. The innovator Brand EK1 achieved a DE of 85.25%. Generic Brand EK4 not only matched this but surpassed it with a value of 87.02%, the highest among the tested brands. This excellent release kinetics reinforced its statistical pharmaceutical equivalence to the innovator brand. Closely aligned, Brand EK2 had DE of 79.31%, a value within the range of the innovator product. However, Brands EK3 and EK7 had lower dissolution efficiencies of 69.79% and 69.52%, respectively. More importantly, the major finding of this study demonstrated that Brands EK5 and EK6, with respective DE of 3.54% and 3.00% consistent with their poor *f*_*1*_ and *f*_*2*_ values, may not be bioequivalent to the innovator brand. Their extreme variation in the model-independent factors reinforces the notion that they may essentially be therapeutically inert.

The *f*_*1*_, *f*_*2*_ and DE data build a solid statistical case indicating that only Brands EK2 and EK4 can be regarded pharmaceutically equivalent to the innovator albendazole 400 mg tablet Brand EK1. Brands EK3 and EK7, while technically meeting compendial specifications, failed the statistical criteria for dissolution similarity and demonstrated moderate DE. Thus, their interchangeability in clinical settings is questionable but borderline acceptable. Contrarily, Brands EK5 and EK6 were spectacular failures, failing to meet compendial specifications by huge margins and collapsing under every statistical and kinetic measure, rendering them unfit for therapeutic substitution. The subpar dissolution profiles exhibited by Brands EK5 and EK6 risks treatment failure and the development of resistant parasite strains. These findings emphasize the necessity of post-market surveillance since not all generic albendazole 400 mg tablet brands available in Nairobi City County, and by extrapolation, Kenyan market, are pharmaceutically equivalent to the innovator brand.

## Discussion

This study demonstrated that although most albendazole 400 mg tablet brands complied with the basic pharmacopoeial specifications, only two generic brands were pharmaceutically equivalent to the innovator product. The findings mirror a global pattern where dissolution emerges as a critical weak link in the quality of albendazole products. In Mexico, Castro et al. (2023) observed that all the three analyzed generic albendazole tablet brands satisfied assay and uniformity of weight test, yet failed dissolution similarity requirements [27]. Results in Yemen paint an even starker scenario where just two in seven brands (29%) were compliant with the dissolution criteria [28]. Similarly, Horton et al. (1999) observed that only one of eight generic albendazole tablet brands (12.5%) matched the innovator in dissolution studies [29]. Ethiopian studies consistently hammer at the same point, where dissolution performance as low as 20% was observed in some albendazole tablet brands, directly shaping treatment outcomes against hookworms [19]. Another study by Mekasha et al. (2024) in the same country found that none of the six tested brands complied with the same critical criteria [18].

Dissolution profile comparison is largely a mandate for biowaivers in generic product development [30]. Consequently, NMRAs such as the United States Food and Drug Administration (US-FDA) and the European Medicines Agency (EMA) have adopted dissolution profile comparison as a surrogate indicator for predictive bioequivalence in the assessment of generic products for marketing authorization through the abbreviated new drug application pathway [31,32]. In this regard, model independent fit factors, including difference factor (*f*_*1*_) and similarity factor (*f*_*2*_), are routinely utilized, alongside model-dependent approaches [33,34]. The US-FDA and EMA recommend *f*_*1*_ and *f*_*2*_ values in the range 0–15 and 50–100, respectively, to infer dissolution profile similarity [35,36]. Being a BCS Class II drug, the gastrointestinal absorption of albendazole and consequently its therapeutic efficacy is strongly retarded by poor aqueous solubility, making dissolution an important surrogate for oral bioavailability. Solid dosage forms that fail dissolution testing are essentially therapeutically inert and culminate in therapeutic failure and contribute to propagation of antimicrobial resistance. The marked variability observed in this study (5.3% to 99.6% released at 60 minutes) strongly suggest differences in therapeutic efficacy. Belew et al. (2015) demonstrated direct correlation between *in vitro* dissolution performance and clinical efficacy outcomes in Ethiopian population, finding that poorly dissolving brands demonstrated reduced cure rates [19].

In the global stage, these results align with the WHO estimates that one in ten medicines in LMICs are substandard or falsified [13]. The study exposes a regulatory blind spot where brands that fail dissolution still show compliance with basic compendial tests, indicating that complying with routine quality checks may not guarantee therapeutic equivalence. Therefore, regulatory authorities should prioritize dissolution profiling, especially for BCS Class II drugs such as albendazole tablets. This will safeguard the therapeutic effectiveness of albendazole tablets, guarantee the success of preventive chemotherapy for STH commonly instituted through population-level and community-based mass drug administration initiatives, and mitigate the risk of antimicrobial resistance.

Albendazole 400 mg tablet brands that failed to comply with the dissolution criteria were also noncompliant with the disintegration specification. This observation is noteworthy, given the longstanding debate surrounding subjecting chewable tablets to disintegration testing. Nevertheless, it is currently agreed that disintegration test is as important to the chewable tablets as it is to the immediate release counterparts. Factors such as wide variations in chewing habits, inadvertent swallowing of whole tablets, and unexpectedly long disintegration times exhibited by some commercial chewable tablets, imply that patient chewing habits alone are unreliable in ensuring proper breakdown of a solid dosage form. Therefore, despite their name, a disintegration test is a critical quality control measure for chewable tablets to ensure consistent drug release and absorption [37].

## Conclusion

Considering that more than a quarter of the world’s population is at risk of infection with the STH [1], it is prudent that available medicines for this scourge remain therapeutically effective to ensure equitable access to healthcare. Albendazole is the cornerstone drug for managing helminth infections in Kenya, yet there is insufficient data on the quality and pharmaceutical equivalence of locally available albendazole products. Given the high prevalence of SF products in developing countries and the deleterious effects they have, including the development of drug resistance, propensity to overdosing and the health burden they cause when treatment fails, it calls for regular assessment of these products to ensure achievement of the United Nations health-related Sustainable Development Goals [38].

This study revealed that, while most albendazole tablet brands complied with basic pharmacopoeial requirements for weight uniformity, friability, hardness and assay, dissolution testing uncovered significant pharmaceutical inequivalence. Only three brands (42.9%), comprising the innovator product and two generic albendazole 400 mg tablet formulations, demonstrated rapid, complete, and statistically similar release kinetics. The other four albendazole tablet brands (57.1%) exhibited slower release rates, with two generic brands (28.6%) being utter failures, releasing negligible amounts of albendazole under the conditions of the test.

The Kenya Pharmacy & Poisons Board, and other NMRAs in the LMICs, should strengthen post-marketing surveillance, emphasizing on dissolution profile analysis considering that compliance with basic pharmacopoeial tests alone does not guarantee therapeutic equivalence. Mass drug administration programs and healthcare providers should use albendazole 400 mg tablet brands that have demonstrable pharmaceutical equivalence to avoid undermining treatment outcomes and inadvertently accelerating emergence and spread of antimicrobial resistance to albendazole and other crucial benzimidazole antiparasitic drugs. Given that this study did not encompass all albendazole 400 mg tablet brands in the market, further studies should be conducted to determine the severity of quality variation in albendazole products.

## Study limitations

The tests conducted were *in vitro* tests which do not necessarily translate to *in vivo* bioequivalence, so the results cannot be directly extrapolated to clinical outcomes. To minimize this, the tests were carried out in simulated gastrointestinal environment at pH 1.2, which closely approximates physiological conditions in the stomach where albendazole is maximally absorbed. Additionally, the sample size did not encompass all the available albendazole 400 mg tablet brands in Kenya, and the findings may not be reflective of the entire Kenyan pharmaceutical market. Nevertheless, the most widely used albendazole 400 mg tablet brands were selected to mitigate this limitation and hence make the sample more representative. Broader surveillance studies are recommended to cover all albendazole 400 mg tablet brands to reach a more inclusive conclusion on the quality of albendazole tablet brands generalizable to the Kenyan pharmaceutical market and the EAC region.
