# Supplementary material for: Post-market surveillance of albendazole tablet brands in Kenya: Implications for deworming programs and clinical outcomes
Source: PLoS Negl Trop Dis. 2026 Jun 16;20(6):e0014449. doi: 10.1371/journal.pntd.0014449 (PMC13286272; doi:10.1371/journal.pntd.0014449)
Supplement: Attachment — Submitted filename: Response to Reviewers.docx [file pntd.0014449.s001.docx]

**MANUSCRIPT ID:	PNTD-D-26-00118**

Thank you for the treasured feedback on our manuscript titled, "**Post-market surveillance of albendazole tablet brands in Kenya: Implications for deworming programs and clinical outcomes**". We greatly appreciate the valuable comments, suggestions, and critique provided by the reviewers that have immensely enriched the manuscript. We have appropriately addressed the reviewers' feedback as described hereafter.

**Methods**

**Reviewer #1**: This paper examines the pharmaceutical characteristics of 7 brands of albendazole on the market in Nairobi, Kenya. The aim was to determine whether such a sample was equivalent to the innovator brand on which all preregistration data was based, and thus be expected to behave similarly. There have been several published studies in the past that have looked at similar samples, and have used generally similar protocols. These are well described in the literature and are the basis of quality control for all approved products. It is regrettable that every study, including this one, over the past 30 years has highlighted the same problem, and yet the same issues arise. The methods described, which are also part of the USP, BP and EUP and the International Pharmacopoeia produced by WHO are adequate to distinguish between products that would be expected to perform like the innovator product and those that would not.

**Response**: We thank the reviewer for the observations regarding the methods used which are similar to other protocols utilized in other studies and commonly prescribed by eminent compendia. We agree with the reviewer that the issues covered in this manuscript are not new. The inadequacy of generic medicines for neglected tropical diseases has been investigated and reported severally. The same issues, including substandard and non-equivalent products that likely deny patients optimal treatment outcomes, have dominated discussions for many years without clear resolution.

**Reviewer #2**: The objectives of the study are clear and accompanied by appropriate study design and methodology. The author should indicate the number of tablets purchased for each brand and the sampling technique that was used. The statistical analysis is appropriate and there are no ethical concerns.

**Response**: We thank the reviewer for bringing to our attention the inadvertent omissions. One-hundred (100) tablets were purchased per brand using convenience sampling technique. This information has been included in the revised manuscript under the subtitle *Sampling technique*.

**Results**

**Reviewer #1**: Clearly presented without any concerns. Tables and figures are fine.

**Response**: We are grateful to the reviewer for this kind observation.

**Reviewer #2**: The results are complete and very well and clearly presented.

**Response**: We appreciate the kind comments by the reviewer.

**Conclusions**

**Reviewer #1**: The conclusions are clear and should be a wake-up call for all Ministries of Health and Pharma Boards, particularly in LMICs. It is essential that all generic products are properly tested, focussing not only on the content of the product but also on its pharmaceutical behaviour. All companies should test dissolution as part of their protocols (both for Registration and QC) and should provide them to the authorities. The authors are aware of this and have the ability to conduct the studies. The same should be true for regulatory bodies, whether using a government or contract lab, and all generic companies should have this available on a continuous basis. The findings in this study are not trivial and poor pharmaceutics can accelerate drug resistance and cause drug failure. Where generics may be important in the public health sector, it is essential that price is not the only consideration when purchasing.

**Response**: We thank the reviewer for these considered observations. Indeed, the National Medicines Regulatory Agencies in the low- & middle-income countries where substandard and falsified health products are prevalent, should be vigilant and ensure that all generic medicines not only comply with pharmacopoeial specifications but also behave similarly to the innovator brands to guarantee achievement of equivalent treatment outcomes notwithstanding the cost of medicines accessible to the patients. This will promote equitable access to quality, safe, and efficacious medicines.

**Reviewer #2**: The results are complete and very well and clearly presented. The authors have clearly described the limitations. The discussion section is concise and very well presented. Public health relevance is clearly articulated in the discussion and conclusion section.

**Response**: The reviewer’s observations are immensely reassuring, and we greatly appreciate them.

**Editorial and data presentation modifications**

**Reviewer #1**: no major issues. the paper is important, if only to continue to highlight the issue.

**Response**: The authors are appreciative of the positive comments by the reviewer. Indeed, the authors couldn't agree more. This work builds on previous studies that have advocated for more stringent postmarketing surveillance of pharmaceutical products to guarantee equitable access to health care for all at all ages, in line with the WHO Sustainable Development Goal 3.

**Reviewer #2**: I recommend some minor revision in the manuscript as follows:

- Change the study area from 'Nairobi' to 'Nairobi City County', for instance in line 23, 26 and 103.

**Response**: Thank you for this recommendation. We have changed the study area to Nairobi City County by revising relevant phrases under the subheadings *Study area* and *Sampling technique*.

- Check whether the dissolution apparatus used is apparatus 1 or 2 as the Pharmacopoeias listed use Dissolution Apparatus 2, while the authors have stated they used apparatus 1. Check line 123.

**Response**: We are grateful to the reviewer for highlighting this error. The study utilized type 2 dissolution apparatus (paddle) as prescribed in the Monograph for albendazole tablets in the United States Pharmacopeia (2025). We have rectified this in the Revised Manuscript under the subheading *Materials, reagents and equipment*.

- Along with the tests carried out, the authors should specify the Pharmacopoeia from which the analytical procedure was adopted, including the acceptance criteria (specifications).

**Response**: We are grateful to the reviewer for this very enriching suggestion, which we have fully implemented in the Revised Manuscript. For each of the seven analytical tests conducted, we have specified the reference pharmacopoeia from which the analytical procedure was adopted, and the acceptance criteria in the Results section.

- The reference Pharmacopoeias listed are not recent. The authors can check the newer editions and compare to see whether any revisions have been carried out. Also there are instances of mix up of editions, for example in line 151 the authors state USP 2018, while in line 241 they state USP 2024.

**Response**: We appreciate these accurate observations. We have checked the newer editions of the reference compendia and confirmed that no revisions have been carried out, implying that we used the most current pharmacopoeial specifications. Nevertheless, we have cited the latest editions of the British Pharmacopoeia (2024), United States Pharmacopeia (2025), and the International Pharmacopoeia (2025), that are within our reach, while avoiding the mix-up with the older editions.

- Since the authors state that they used the USP for dissolution testing, it is important to note that the USP states the dissolution should be carried using apparatus 2 at 50 RPM for 30 minutes and the acceptance criteria is NLT 80%(Q). However, the International Pharmacopoeia (Ph. Int.) uses the same conditions but at 75 RPM. Therefore, the authors can change the cited pharmacopoeia from USP to Ph. Int. Indeed, the Ph. Int. is very specific for albendazole chewable tablets, while the USP is for general for albendazole tablets.

**Response**: The authors are grateful to the reviewer for this suggestion. We agree that USP specifies the use of Dissolution Apparatus 2 (paddle) for albendazole tablets. A typographical error in Methods section reported that this study used Dissolution Apparatus 1, while in actual fact, we used Apparatus 2. For Dissolution Apparatus 2, the USP specifies that the paddle should be rotated at 75 revolutions per minute for albendazole tablets. This is well reflected in the Revised Manuscript.

- The authors should also calculate the amount of albendazole dissolved at 30 minutes and determine which brands comply with the specifications for dissolution testing. They then can use the 60 minutes for comparison of dissolution profiles using the similarity factors.

**Response**: We thank the reviewer for this suggestion, which we have fully implemented in the revised manuscript by presenting results for dissolution testing and pharmaceutical equivalence separately. We have used the dissolution data at 30 min to evaluate compliance with the United States Pharmacopeia (2025) specification for dissolution test (Table 2). The interpretations are incorporated into the *Dissolution testing* section of the Revised manuscript. On the other hand, the data relevant to pharmaceutical equivalence are presented separately using dissolution data for the entire 60 minutes. The results are contextualized in the literature by providing a brief overview of the importance of dissolution profile comparison in the *Discussion* section (Paragraph 2), accompanied by relevant citations. We believe that the presentation is satisfactory and appreciate the reviewers for this enriching suggestion.

- The authors should state the importance of chewing and whether disintegration test is an appropriate test for chewable tablets.

**Response**: We thank the reviewer for raising this query. Indeed, there have been longstanding debates surrounding subjecting chewable tablets to disintegration testing. Nevertheless, it is currently agreed that disintegration test is as important to the chewable tablets as it is to the immediate release counterparts. According to recent guidance by the Food & Drug Administration, some commercial chewable tablets exhibit unexpectedly long disintegration times, emphasizing that patient chewing habits alone are unreliable for ensuring proper drug breakdown. Therefore, despite their name, a disintegration test is a critical quality control measure for chewable tablets to ensure consistent drug release and absorption. We have included this information in the last paragraph of the *Discussion* section.

**Summary and General Comments**

**Reviewer #1**: Some minor points:

- I could not find any indication of tablet strength in the paper. Were they 200mg or 400mg? line 143-4 this is not clear to me. 20 x200mg tablets would be 4.0g powder?

**Response**: The authors gratefully appreciate the reviewer for bringing this omission to their attention. The study evaluated albendazole 400 mg tablet brands as had been noted in the study objective. However, we have now made revisions throughout the manuscript to explicitly emphasize the study focus on albendazole 400 mg tablets. Revisions have been made in the relevant phrases as appropriate in the *Introduction*, *Experimental sections*, *Results*, *Discussion*, and *Conclusion* to correct the identified ambiguity.

In this case, therefore, 20 × 400 mg tablets provided a powder equivalent to 8.0 g of albendazole.

- Line 303 it should be EK6?

**Response**: We thank the reviewer for pointing out this typographical error. Yes, indeed. The albendazole 400 mg tablet brand should be EK6 and not EK7. We have rectified this accordingly in the Revised Manuscript.

- Line 308 it should be pharmaceutically equivalent. Bioequivalence refers to PK equivalence.

**Response**: The authors oblige this correction; we totally agree that the wording here should refer to pharmaceutical equivalence and not bioequivalence since the study did not involve pharmacokinetics analysis. We have therefore revised the phrase to read ‘pharmaceutically equivalent’.

- line 325 Horton et al not in references. it is 65. Gallia, E., Horton, J., Dressman, JB. (1999) Albendazole Generics- a comparative in vitro study. Pharmaceutical Research. 16, 1871-1875.

**Response**: We appreciate the reviewer for bringing this omission to our attention, and for providing the citation. We have subsequently included it in the List of References (Ref.?).

**Reviewer #2**: The manuscript is adequate in form and content and I recommend its publication after minor revision. The work provides additional data on quality of albendazole tablets in the Kenyan market and its implication on public health and the Kenyan healthcare system.

**Response**: The authors greatly appreciate the positive assessment made by the reviewer. We are honored that this piece of work is recognized as a significant contribution to improved healthcare provision in Kenya.
