# Supplementary material for: Post-market surveillance of albendazole tablet brands in Kenya: Implications for deworming programs and clinical outcomes
Source: PLoS Negl Trop Dis. 2026 Jun 16;20(6):e0014449. doi: 10.1371/journal.pntd.0014449 (PMC13286272; doi:10.1371/journal.pntd.0014449)
Supplement: Peer review history

# Peer review: Decision Letter 0

**DOI:** 10.1371/journal.pntd.0014449.r001

**Authors:** Sabine Specht, Lucienne Tritten

23 Apr 2026

PNTD-D-26-00118

Post-market surveillance of albendazole tablet brands in Kenya: Implications for deworming programs and clinical outcomes

PLOS Neglected Tropical Diseases

Dear Dr. Peter Mbugua Njogu,

Thank you for submitting your manuscript to PLOS Neglected Tropical Diseases. After careful consideration, we feel that it has merit but does not fully meet PLOS Neglected Tropical Diseases’s publication criteria as it currently stands. Therefore, we invite you to submit a revised version of the manuscript that addresses the points raised during the review process.

Please submit your revised manuscript by May 22nd. If you will need more time than this to complete your revisions, please reply to this message or contact the journal office at plosntds@plos.org. When you're ready to submit your revision, log on to https://www.editorialmanager.com/pntd/ and select the ’submissions Needing Revision' folder to locate your manuscript file.

Please include the following items when submitting your revised manuscript:

* A letter that responds to each point raised by the editor and reviewer(s). You should upload this letter as a separate file labeled 'Response to Reviewers'. This file does not need to include responses to any formatting updates and technical items listed in the 'Journal Requirements' section below.

* A marked-up copy of your manuscript that highlights changes made to the original version. You should upload this as a separate file labeled 'Revised Manuscript with Track Changes'.

* An unmarked version of your revised paper without tracked changes. You should upload this as a separate file labeled 'Manuscript'.

If you would like to make changes to your financial disclosure, competing interests statement, or data availability statement, please make these updates within the submission form at the time of resubmission. Guidelines for resubmitting your figure files are available below the reviewer comments at the end of this letter.

We look forward to receiving your revised manuscript.

Kind regards,

Sabine Specht

Academic Editor

PLOS Neglected Tropical Diseases

Lucienne Tritten

Section Editor

PLOS Neglected Tropical Diseases

Shaden Kamhawi

co-Editor-in-Chief

PLOS Neglected Tropical Diseases

orcid.org/0000-0003-4304-636XX

Paul Brindley

co-Editor-in-Chief

PLOS Neglected Tropical Diseases

orcid.org/0000-0003-1765-0002

**Journal Requirements:**

1) Please ensure that the CRediT author contributions listed for every co-author are completed accurately and in full.

At this stage, the following Authors/Authors require contributions: Peter Mbugua Njogu. Please ensure that the full contributions of each author are acknowledged in the "Add/Edit/Remove Authors" section of our submission form.

The list of CRediT author contributions may be found here: https://journals.plos.org/plosntds/s/authorship#loc-author-contributions

2) Please provide an Author Summary. This should appear in your manuscript between the Abstract (if applicable) and the Introduction, and should be 150-200 words long. The aim should be to make your findings accessible to a wide audience that includes both scientists and non-scientists. Sample summaries can be found on our website under Submission Guidelines:

https://journals.plos.org/plosntds/s/submission-guidelines#loc-parts-of-a-submission

3) We do not publish any copyright or trademark symbols that usually accompany proprietary names, eg ©,  ®, or TM  (e.g. next to drug or reagent names). Therefore please remove all instances of trademark/copyright symbols throughout the text, including:

- ® on pages: 2, 5, and 12.

4) Please upload all main figures as separate Figure files in .tif or .eps format. For more information about how to convert and format your figure files please see our guidelines:

https://journals.plos.org/plosntds/s/figures

5) Tables should not be uploaded as individual files. Please remove these files and include the Tables in your manuscript file as editable, cell-based objects. For more information about how to format tables, see our guidelines:

https://journals.plos.org/plosntds/s/tables

**Reviewers' comments:**

Reviewer’s Responses to Questions

**Key Review Criteria Required for Acceptance?**

As you describe the new analyses required for acceptance, please consider the following:

**Methods:**

-Are the objectives of the study clearly articulated with a clear testable hypothesis stated?

-Is the study design appropriate to address the stated objectives?

-Is the population clearly described and appropriate for the hypothesis being tested?

-Is the sample size sufficient to ensure adequate power to address the hypothesis being tested?

-Were correct statistical analysis used to support conclusions?

-Are there concerns about ethical or regulatory requirements being met?

Reviewer #1: This paper examines the pharmaceutical characteristics of 7 brands of albendazole on the market in Nairobi, Kenya. The aim was to determine whether such a sample was equivalent to the innovator brand on which all pre-registration data was based, and thus be expected to behave similarly. There have been several published studies in the past that have looked at similar samples, and have used generally similar protocols. These are well described in the literature and are the basis of quality control for all approved products. It is regrettable that every study, including this one, over the past 30 years has highlighted the same problem, and yet the same issues arise.

The methods described, which are also part of the USP, BP and EUP and the International Pharmacopoeia produced by WHO are adequate to distinguish between products that would be expected to perform like the innovator product and those that would not.

Reviewer #2: The objectives of the study are clear and accompanied by appropriate study design and methodology.

The author should indicate the number of tablets purchased for each brand and the sampling technique that was used.

The statistical analysis is appropriate and there are no ethical concerns.

**********

**Results**

-Does the analysis presented match the analysis plan?

-Are the results clearly and completely presented?

-Are the figures (Tables, Images) of sufficient quality for clarity?

Reviewer #1: Clearly presented without an concerns. Tables and figures are fine.

Reviewer #2: The results are complete and very well and clearly presented.

**********

**Conclusions**

-Are the conclusions supported by the data presented?

-Are the limitations of analysis clearly described?

-Do the authors discuss how these data can be helpful to advance our understanding of the topic under study?

-Is public health relevance addressed?

Reviewer #1: The conclusions are clear and should be a wake-up call for all Ministries of Health and Pharma Boards, particularly in LMICs. It is essential that all generic products are properly tested, focussing not only on the content of the product but also on its pharmaceutical behaviour. All companies should test dissolution as part of their protocols (both for Registration and QC) and should provide them to the authorities. The authors are aware of this and have the ability to conduct the studies. The same should be true for regulatory bodies, whether using a government or contract lab, and all generic companies should have this available on a continuous basis.

The findings in this study are not trivial and poor pharmaceutics can accelerate drug resistance and cause drug failure. Where generics may be important in the public health sector, it is essential that price is not the only consideration when purchasing.

Reviewer #2: The conclusions align very well with results.

The authors have clearly described the limitations.

The discussion section is concise and very well presented.

Public health relevance is clearly articulated in the discussion and conclusion section.

**********

**Editorial and Data Presentation Modifications?**

Use this section for editorial suggestions as well as relatively minor modifications of existing data that would enhance clarity. If the only modifications needed are minor and/or editorial, you may wish to recommend “Minor Revision” or “Accept”.

Reviewer #1: no major issues. the paper is important, if only to continue to highlight the issue.

Reviewer #2: I recommend some minor revision in the manuscript as follows:

- Change the study area from 'Nairobi' to 'Nairobi City County', for instance in line 23, 26 and 103.

- Check whether the dissolution apparatus used is apparatus 1 or 2 as the Pharmacopoeias listed use Dissolution Apparatus 2, while the authors have stated they used apparatus 1. Check line 123.

- Along with the tests carried out, the authors should specify the Pharmacopoeia from which the analytical procedure was adopted, including the acceptance criteria (specifications).

- The reference Pharmacopoeias listed are not recent. The authors can check the newer editions and compare to see whether any revisions have been carried out. Also there are instances of mix up of editions, for example in line 151 the authors state USP 2018, while in line 241 they state USP 2024.

- Since the authors state that they used the USP for dissolution testing, it is important to note that the USP states the dissolution should be carried using apparatus 2 at 50 RPM for 30 minutes and the acceptance criteria is NLT 80%(Q). However, the International Pharmacopoeia (Ph. Int.) uses the same conditions but at 75 RPM. Therefore, the authors can change the cited pharmacopoeia from USP to Ph. Int. Indeed, the Ph. Int. is very specific for albendazole chewable tablets, while the USP is for general for albendazole tablets.

- The authors should also calculate the amount of albendazole dissolved at 30 minutes and determine which brands comply with the specifications for dissolution testing. They then can use the 60 minutes for comparison of dissolution profiles using the similarity factors.

- The authors should state the importance of chewing and whether disintegration test is an appropriate test for chewable tablets.

**********

**Summary and General Comments**

Use this section to provide overall comments, discuss strengths/weaknesses of the study, novelty, significance, general execution and scholarship. You may also include additional comments for the author, including concerns about dual publication, research ethics, or publication ethics. If requesting major revision, please articulate the new experiments that are needed.

Reviewer #1: Some minor points.

I could not find any indication of tablet strength in the paper. Were they 200mg or 400mg?

line 143-4 this is not clear to me. 20 x200mg tablets would be 4.0g powder?

Line 303 it should be EK6?

Line 308 it should be pharmaceutically equivalent. Bioequivalence refers to PK equivalence

line 325 Horton et al not in references. it is 65. Gallia, E., Horton, J., Dressman, JB. (1999) Albendazole Generics – a comparative in vitro study. Pharmaceutical Research. 16, 1871-1875

AN EXCELLENT PAPER.

Reviewer #2: The manuscript is adequate in form and content and I recommend its publication after minor revision. The work provides additional data on quality of albendazole tablets in the Kenyan market and its implication on public health and the Kenyan healthcare system.

**********

PLOS authors have the option to publish the peer review history of their article (what does this mean?). If published, this will include your full peer review and any attached files.

If you choose “no”, your identity will remain anonymous but your review may still be made public.

**Do you want your identity to be public for this peer review?**   For information about this choice, including consent withdrawal, please see our Privacy Policy.

Reviewer #1: **Yes:**   John Horton

Reviewer #2: **Yes:**   Dr Paul Njaria

[NOTE: If reviewer comments were submitted as an attachment file, they will be attached to this email and accessible via the submission site. Please log into your account, locate the manuscript record, and check for the action link "View Attachments". If this link does not appear, there are no attachment files.]

**Figure resubmission:**

While revising your submission, we strongly recommend that you use PLOS’s NAAS tool (https://ngplosjournals.pagemajik.ai/artanalysis) to test your figure files. NAAS can convert your figure files to the TIFF file type and meet basic requirements (such as print size, resolution), or provide you with a report on issues that do not meet our requirements and that NAAS cannot fix.

After uploading your figures to PLOS’s NAAS tool - https://ngplosjournals.pagemajik.ai/artanalysis, NAAS will process the files provided and display the results in the "Uploaded Files" section of the page as the processing is complete. If the uploaded figures meet our requirements (or NAAS is able to fix the files to meet our requirements), the figure will be marked as "fixed" above. If NAAS is unable to fix the files, a red "failed" label will appear above. When NAAS has confirmed that the figure files meet our requirements, please download the file via the download option, and include these NAAS processed figure files when submitting your revised manuscript.

**Reproducibility:**

To enhance the reproducibility of your results, we recommend that authors of applicable studies deposit laboratory protocols in protocols.io, where a protocol can be assigned its own identifier (DOI) such that it can be cited independently in the future. Additionally, PLOS ONE offers an option to publish peer-reviewed clinical study protocols. Read more information on sharing protocols at https://plos.org/protocols?utm_medium=editorial-email&utm_source=authorletters&utm_campaign=protocols

# Author comment: Author response to Decision Letter 1

**DOI:** 10.1371/journal.pntd.0014449.r002

17 May 2026

Attachment Submitted filename: Response to Reviewers.docx

# Editor report: Decision Letter 1

**DOI:** 10.1371/journal.pntd.0014449.r003

**Authors:** Sabine Specht, Lucienne Tritten

8 Jun 2026

Dear Dr Njogu,

We are pleased to inform you that your manuscript 'Post-market surveillance of albendazole tablet brands in Kenya: Implications for deworming programs and clinical outcomes' has been provisionally accepted for publication in PLOS Neglected Tropical Diseases.

Before your manuscript can be formally accepted you will need to complete some formatting changes, which you will receive in a follow up email. A member of our team will be in touch with a set of requests.

Please note that your manuscript will not be scheduled for publication until you have made the required changes, so a swift response is appreciated.

IMPORTANT: The editorial review process is now complete. PLOS will only permit corrections to spelling, formatting or significant scientific errors from this point onwards. Requests for major changes, or any which affect the scientific understanding of your work, will cause delays to the publication date of your manuscript.

Should you, your institution’s press office or the journal office choose to press release your paper, you will automatically be opted out of early publication. We ask that you notify us now if you or your institution is planning to press release the article. All press must be co-ordinated with PLOS.

Thank you again for supporting Open Access publishing; we are looking forward to publishing your work in PLOS Neglected Tropical Diseases.

Best regards,

Sabine Specht

Academic Editor

PLOS Neglected Tropical Diseases

Lucienne Tritten

Section Editor

PLOS Neglected Tropical Diseases

Shaden Kamhawi

co-Editor-in-Chief

PLOS Neglected Tropical Diseases

orcid.org/0000-0003-4304-636XX

Paul Brindley

co-Editor-in-Chief

PLOS Neglected Tropical Diseases

orcid.org/0000-0003-1765-0002

***********************************************************

p.p1 {margin: 0.0px 0.0px 0.0px 0.0px; line-height: 16.0px; font: 14.0px Arial; color: #323333; -webkit-text-stroke: #323333}span.s1 {font-kerning: none

# Editor report: Acceptance letter

**DOI:** 10.1371/journal.pntd.0014449.r004

**Authors:** Sabine Specht, Lucienne Tritten

Dear Dr. Njogu,

We are delighted to inform you that your manuscript, "Post-market surveillance of albendazole tablet brands in Kenya: Implications for deworming programs and clinical outcomes," has been formally accepted for publication in PLOS Neglected Tropical Diseases.

We have now passed your article onto the PLOS Production Department who will complete the rest of the publication process. All authors will receive a confirmation email upon publication.

The corresponding author will soon be receiving a typeset proof for review, to ensure errors have not been introduced during production. Please review the PDF proof of your manuscript carefully, as this is the last chance to correct any scientific or type-setting errors. Please note that major changes, or those which affect the scientific understanding of the work, will likely cause delays to the publication date of your manuscript. Note: Proofs for Front Matter articles (Editorial, Viewpoint, Symposium, Review, etc...) are generated on a different schedule and may not be made available as quickly.

Soon after your final files are uploaded, the early version of your manuscript will be published online unless you opted out of this process. The date of the early version will be your article’s publication date. The final article will be published to the same URL, and all versions of the paper will be accessible to readers.

For Research Articles, you will receive an invoice from PLOS for your publication fee after your manuscript has reached the completed accept phase. If you receive an email requesting payment before acceptance or for any other service, this may be a phishing scheme. Learn how to identify phishing emails and protect your accounts at https://explore.plos.org/phishing.

Thank you again for supporting open-access publishing; we are looking forward to publishing your work in PLOS Neglected Tropical Diseases.

Best regards,

Shaden Kamhawi

co-Editor-in-Chief

PLOS Neglected Tropical Diseases

Paul Brindley

co-Editor-in-Chief

PLOS Neglected Tropical Diseases
